# Supplementary material for: Epidemiology of intestinal parasite infections and multiparasitism and their impact on growth and hemoglobin levels during childhood in tropical Ecuador: A longitudinal study using molecular detection methods
Source: PLoS Negl Trop Dis. 2025 Jun 16;19(6):e0013004. doi: 10.1371/journal.pntd.0013004 (PMC12169531; doi:10.1371/journal.pntd.0013004)
Supplement: S1 Table — Summaries are made upon ever/never infected to preserve the independence of the observations. Infection frequencies represent any infection identified during follow-up from 7 months to 8 years of age. Frequencies for categories of time-varying variables represent data recorded at birth. Characteristics are at time of birth of child or time-varying (TV) over the course of follow-up. STH—soil-transmitted helminth infections. Afro- Afro-Ecuadorian. Anthelmintic treatments—maternal reports of at least one anthelmintic treatment during the previous year. Monthly household income was classified according to receipt of an income sufficient to meet the basic needs of 4 persons (or 1 family basket) or US$480 in 2008. Household overcrowding was defined as 3 or more people per sleeping room. Agricultural exposures were defined by living on a farm or having at least weekly visits to a farm. Any infected with STH in the household represented any member of the child’s household with a positive stool sample collected around the time of birth of the cohort child. (DOCX) [file pntd.0013004.s001.docx]

| **Variables** | **Categories** | **Any STH** | | **Any protozoa** | | **Any parasite** | | **TOTAL** |
| --- | --- | --- | --- | --- | --- | --- | --- | --- |
|  |  | **Negative** | **Ever Positive** | **Negative** | **Ever Positive** | **Negative** | **Ever Positive** |  |
|  |  | **194 (48.38%)** | **207(51.62%)** | **51(12.72%)** | **350(87.28%)** | **35(8.73%)** | **366(91.27%)** | **401** |
| **CHILDHOOD FACTORS** |  |  |  |  |  |  |  |  |
| **Sex** | **Male** | 113 (52.07%) | 104 (47.93%) | 27 (12.44%) | 190 (87.56%) | 23 (10.60%) | 194 (89.40%) | 217 |
|  | **Female** | 81 (44.02%) | 103 (55.98%) | 24 (13.04%) | 160 (86.96%) | 12 (6.52%) | 172 (93.48%) | 184 |
| **Birth order** | **1^st^-2^nd^** | 118 (55.40%) | 95 (44.60%) | 33 (15.49%) | 180 (84.51%) | 23 (10.80%) | 190 (89.20%) | 213 |
|  | **3^rd^-4^th^** | 47 (40.17%) | 70 (59.83%) | 11 (9.40%) | 106 (90.60%) | 6 (5.13%) | 111 (94.87%) | 117 |
|  | **≥5^th^** | 29 (40.85%) | 42 (59.15%) | 7 (9.86%) | 64 (90.14%) | 6 (8.45%) | 65 (91.55%) | 71 |
| **Delivery mode** | **Vaginal** | 131 (43.67%) | 169 (56.33%) | 33 (11.00%) | 267 (89.00%) | 22 (7.33%) | 278 (92.67%) | 300 |
|  | **Caesarean** | 63 (62.38%) | 38 (37.62%) | 18 (17.82%) | 83 (82.18%) | 13 (12.87%) | 88 (87.13%) | 101 |
| **Breastfeeding (months)** | **0-6** | 21 (61.76%) | 13 (38.24%) | 6 (17.65%) | 28 (82.35%) | 6 (17.65%) | 28 (82.35%) | 34 |
|  | **7-12** | 88 (47.31%) | 98 (52.69%) | 21 (11.29%) | 165 (88.71%) | 14 (7.53%) | 172 (92.47%) | 186 |
|  | **>12** | 75 (46.01%) | 88 (53.99%) | 20 (12.27%) | 143 (87.73%) | 11 (6.75%) | 152 (93.25%) | 163 |
|  | **Missing** | 10 (55.56%) | 8 (44.44%) | 4 (22.22%) | 14 (77.78%) | 4 (22.22%) | 14 (77.78%) | 18 |
| **Exclusive breastfeeding** | **Mean/SD** | 3.39/2.61 | 3.72/2.37 | 2.86/2.48 | 3.67/2.47 | 2.74/2.45 | 3.64/2.48 |  |
| **(months)** | **Median (Q1-Q3)** | 4(1, 6) | 4(2, 6) | 3(0, 5) | 4(1, 6) | 3(0, 5) | 4(1, 6) |  |
| **Day care to 3 years** | **No** | 170 (50.30%) | 168 (49.70%) | 46 (13.61%) | 292 (86.39%) | 31 (9.17%) | 307 (90.83%) | 338 |
|  | **Yes** | 24 (38.10%) | 39 (61.90%) | 5 (7.94%) | 58 (92.06%) | 4 (6.35%) | 59 (93.65%) | 63 |
| **Anthelmintic treatment** | **Never** | 6(3.1%) | 1(0.5%) | 3 (5.9%) | 4(1.4%) | 3 (8.6%) | 4(1.1%) | 7(1.8%) |
| **(TV)** | **Ever** | 188(96.9%) | 206(99.5%) | 48(94.1%) | 346(98.9%) | 32(91.4%) | 362(98.9%) | 394(98.3%) |
| **MATERNAL FACTORS** |  |  |  |  |  |  |  |  |
| **Age (years)** | **≤20** | 59 (52.68%) | 53 (47.32%) | 13 (11.61%) | 99 (88.39%) | 9 (8.04%) | 103 (91.96%) | 112 |
|  | **21-29** | 90 (46.39%) | 104 (53.61%) | 23 (11.86%) | 171 (88.14%) | 15 (7.73%) | 179 (92.27%) | 194 |
|  | **≥30** | 45 (47.37%) | 50 (52.63%) | 15 (15.79%) | 80 (84.21%) | 11 (11.58%) | 84 (88.42%) | 95 |
| **Ethnicity** | **Afro** | 39 (34.51%) | 74 (65.49%) | 12 (10.62%) | 101 (89.38%) | 8 (7.08%) | 105 (92.92%) | 113 |
|  | **Non-Afro** | 155 (53.82%) | 133 (46.18%) | 39 (13.54 %) | 249 (86.46 %) | 27 (9.38%) | 261 (90.63%) | 288 |
| **Educational status** | **Illiterate** | 22 (43.14%) | 29 (56.86%) | 4 (7.84%) | 47 (92.16%) | 3 (5.88%) | 48 (94.12%) | 51 |
|  | **Primary** | 109 (44.67%) | 135 (55.33%) | 35 (14.34%) | 209 (85.66%) | 22 (9.02%) | 222 (90.98%) | 244 |
|  | **Secondary** | 63 (59.43%) | 43 (40.57%) | 12 (11.32%) | 94 (88.68 %) | 10 (9.43%) | 96 (90.57%) | 106 |
| **HOUSEHOLD FACTORS** |  |  |  |  |  |  |  |  |
| **Socio-economic status** | **Low** | 70 (47.62%) | 77 (52.38%) | 17 (11.56%) | 130 (88.44%) | 11 (7.48%) | 136 (92.52%) | 147 |
|  | **Medium** | 51 (39.84%) | 77 (60.16%) | 22 (17.19%) | 106 (82.81%) | 13 (10.16%) | 115 (89.84%) | 128 |
|  | **High** | 73 (57.94%) | 53 (42.06%) | 12 (9.52%) | 114 (90.48%) | 11 (8.73%) | 115 (91.27%) | 126 |
| **Area of residence** | **Urban** | 139 (51.10%) | 133 (48.90%) | 32 (11.76%) | 240 (88.24%) | 26 (9.56%) | 246 (90.44%) | 272 |
|  | **Rural** | 55 (42.64%) | 74 (57.36%) | 19 (14.73%) | 110 (85.27%) | 9 (6.98%) | 120 (93.02%) | 129 |
| **Overcrowding** | **<3** | 121 (52.38%) | 110 (47.62%) | 32 (13.85%) | 199 (86.15%) | 23 (9.96%) | 208 (90.04%) | 231 |
|  | **≥3** | 73 (42.94%) | 97 (57.06%) | 19 (11.18%) | 151 (88.82%) | 12 (7.06%) | 158 (92.94%) | 170 |
| **Monthly income** | **< 1 basket** | 157 (46.18%) | 183 (53.82%) | 38 (11.18%) | 302 (88.82%) | 26 (7.65%) | 314 (92.35%) | 340 |
|  | **≥ 1 basket** | 14 (77.78%) | 4 (22.22%) | 5 (27.78%) | 13 (72.22%) | 4 (22.22%) | 14 (77.78%) | 18 |
|  | **Missing** | 23 (53.49%) | 20 (46.51%) | 8 (18.60%) | 35 (81.40%) | 5 (11.63%) | 38 (88.37%) | 43 |
| **House construction** | **Wood/bamboo** | 55 (47.01%) | 62 (52.99%) | 14 (11.97%) | 103 (88.03%) | 8 (6.84%) | 109 (93.16%) | 117 |
|  | **Cement/brick** | 139 (49.64%) | 141 (50.36%) | 37 (13.21%) | 243 (86.79%) | 27 (9.64%) | 253 (90.36%) | 280 |
|  | **Missing** | 0 (0.00%) | 4 (100.00%) | 0 (0.00%) | 4 (100.00%) | 0 (0.00%) | 4 (100.00%) | 4 |
| **Dog in house (TV)** | **No** | 173 (48.87%) | 181 (51.13%) | 43 (12.15%) | 311 (87.85%) | 31 (8.76%) | 323 (91.24%) | 354 |
|  | **Yes** | 21 (44.68%) | 26 (55.32%) | 8 (17.02%) | 39 (82.98%) | 4 (8.51%) | 43 (91.49%) | 47 |
| **Cat in house (TV)** | **No** | 175 (49.86%) | 176 (50.14%) | 41 (11.68%) | 310 (88.32%) | 29 (8.26%) | 322 (91.74%) | 351 |
|  | **Yes** | 19 (38.00%) | 31 (62.00%) | 10 (20.00%) | 40 (80.00%) | 6 (12.00%) | 44 (88.00%) | 50 |
| **Agriculture (TV)** | **No** | 109 (57.98%) | 79 (42.02%) | 23 (12.23%) | 165 (87.77%) | 19 (10.11%) | 169 (89.89%) | 188 |
|  | **Yes** | 85 (39.91%) | 128 (60.09%) | 28 (13.15%) | 185 (86.85%) | 16 (7.51%) | 197 (92.49%) | 213 |
| **Bathroom (TV)** | **Latrine** | 17 (58.62%) | 12 (41.38%) | 10 (34.48%) | 19 (65.52%) | 10 (34.48%) | 19 (65.52%) | 29 |
|  | **WC** | 177 (47.58%) | 195 (52.42%) | 41 (11.02%) | 331 (88.98%) | 25 (6.72%) | 347 (93.28%) | 372 |
| **HOUSEHOLD STH INFECTIONS** |  |  |  |  |  |  |  |  |
| **Maternal** | **No** | 145 (50.70%) | 141 (49.30%) | 43 (15.03%) | 243 (84.97%) | 29 (10.14%) | 257 (89.86%) | 286 |
|  | **Yes** | 49 (42.61%) | 66 (57.39%) | 8 (6.96%) | 107 (93.04%) | 6 (5.22%) | 109 (94.78%) | 115 |
| **Paternal** | **No** | 173 (48.87%) | 181 (51.13%) | 45 (12.71%) | 309 (87.29%) | 30 (8.47%) | 324 (91.53%) | 354 |
|  | **Yes** | 21 (44.68%) | 26 (55.32%) | 6 (12.77%) | 41 (87.23%) | 5 (10.64%) | 42 (89.36%) | 47 |
| **Siblings** | **No** | 155 (51.16%) | 148 (48.84%) | 44 (14.52%) | 259 (85.48%) | 31 (10.23%) | 272 (89.77%) | 303 |
|  | **Yes** | 39 (39.80%) | 59 (60.20%) | 7 (7.14%) | 91 (92.86%) | 4 (4.08%) | 94 (95.92%) | 98 |
| **Any in household** | **No** | 137 (51.31%) | 130 (48.69%) | 39 (14.61%) | 228 (85.39%) | 28 (10.49%) | 239 (89.51%) | 267 |
|  | **Yes** | 57 (42.54%) | 77 (57.46%) | 12 (8.96%) | 122 (91.04%) | 7 (5.22%) | 127 (94.78%) | 134 |

**S1 Table. Descriptive statistics for frequences of infections with any STH, any protozoa, and any parasite in 401 children according to categories of childhood, maternal, and household factors. Summaries are made upon ever/never infected to preserve the independence of the observations. Infection frequencies represent any infection identified during follow-up from 7 months to 8 years of age. Frequencies for categories of time-varying variables represent data recorded at birth.**

Characteristics are at time of birth of child or time-varying (TV) over the course of follow-up. STH—soil-transmitted helminth infections. Afro- Afro-Ecuadorian. Anthelmintic treatments—maternal reports of at least one anthelmintic treatment during the previous year. Monthly household income was classified according to receipt of an income sufficient to meet the basic needs of 4 persons (or 1 family basket) or US$480 in 2008. Household overcrowding was defined as 3 or more people per sleeping room. Agricultural exposures were defined by living on a farm or having at least weekly visits to a farm. Any infected with STH in the household represented any member of the child’s household with a positive stool sample collected around the time of birth of the cohort child.
