# Supplementary material for: Epidemiology of intestinal parasite infections and multiparasitism and their impact on growth and hemoglobin levels during childhood in tropical Ecuador: A longitudinal study using molecular detection methods
Source: PLoS Negl Trop Dis. 2025 Jun 16;19(6):e0013004. doi: 10.1371/journal.pntd.0013004 (PMC12169531; doi:10.1371/journal.pntd.0013004)
Supplement: S2 Table — Odds ratios (ORs) and 95% confidence intervals (CI) were estimated by fitting age, age2, and age3-adjusted longitudinal models using generalized estimating equations. Longitudinal binary outcomes were defined by presence/absence of any STH or any protozoa detected in stool samples from children during follow-up. Models were fit under missing completely at random assumption for unobserved data points. Characteristics are at time of birth of child (birth) or time-varying (TV) over the course of follow-up. STH—soil-transmitted helminth infections. Afro- Afro-Ecuadorian. Anthelmintic treatments—maternal reports of at least one anthelmintic treatment during the previous year. Monthly household income was classified according to receipt of an income sufficient to meet the basic needs of 4 persons (or 1 family basket) or US$480 in 2008. Household overcrowding was defined as 3 or more people per sleeping room. Agricultural exposures were defined by living on a farm or having at least weekly visits to a farm. Any infected with STH in the household represented any member of the child’s household with a positive stool sample collected around the time of birth of the cohort child. (DOCX) [file pntd.0013004.s002.docx]

| **Variables** | **Categories** | **Any STH** | | | | **Any protozoa** | | | |
| --- | --- | --- | --- | --- | --- | --- | --- | --- | --- |
|  |  | **OR** | **P-value** | **95%CIlow** | **95%CI high** | **OR** | **P-value** | **95%CI low** | **95%CI high** |
| **CHILDOOD FACTORS** |  |  |  |  |  |  |  |  |  |
| **AGE** |  | 1.263 | **<0.001** | 1.187 | 1.343 | 2.622 | **<0.001** | 1.596 | 4.309 |
| **AGE^2^** |  | 0.996 | **<0.001** | 0.994 | 0.997 | 0.949 | **0.004** | 0.916 | 0.983 |
| **AGE^3^** |  | 1.0002 | **0.011** | 1.000 | 1.0003 | 1.0001 | **0.014** | 1.0000 | 1.0002 |
| **SEX** | **GIRLS vs. BOYS** | 1.183 | 0.182 | 0.925 | 1.512 | 0.704 | **0.001** | 0.574 | 0.862 |
| **BIRTH ORDER** | **3^rd^-4^th^ vs. 1^st^-2^nd^** | 1.670 | **<0.001** | 1.260 | 2.213 | 1.280 | **0.036** | 1.017 | 1.612 |
|  | **≥5^th^ vs. 1^st^-2^nd^** | 2.059 | **<0.001** | 1.482 | 2.859 | 1.529 | **0.003** | 1.157 | 2.021 |
| **DELIVERY MODE** | **Vaginal vs. Caesarean** | 0.554 | **<0.001** | 0.406 | 0.755 | 0.726 | **0.008** | 0.574 | 0.919 |
| **EXCLUSIVE BREASTFEEDING** | **1 MONTH EFFECT** | 1.625 | **0.003** | 1.178 | 2.241 | 1.020 | 0.315 | 0.981 | 1.062 |
| **DAY CARE TO 3 years** | **YES vs. NO** | 1.635 | **0.028** | 1.055 | 2.534 | 1.953 | **<0.001** | 1.473 | 2.590 |
| **ANTHELMINTICS (TV)** | **YES vs. NO** | 0.923 | 0.651 | 0.653 | 1.305 | 0.897 | 0.446 | 0.677 | 1.187 |
| **MATERNAL FACTORS** |  |  |  |  |  |  |  |  |  |
| **AGE GROUP** | **21-29 vs. ≤20** | 1.090 | 0.573 | 0.808 | 1.471 | 0.901 | 0.400 | 0.706 | 1.149 |
|  | **≥30 vs. ≤20** | 0.935 | 0.706 | 0.660 | 1.326 | 0.779 | 0.084 | 0.587 | 1.034 |
| **ETHNICITY** | **NON_AFRO vs. AFRO** | 0.415 | **<0.001** | 0.319 | 0.541 | 0.702 | **0.002** | 0.561 | 0.879 |
|  | **(NON_AFRO vs. AFRO) ×AGE** |  |  |  |  |  |  |  |  |
| **EDUCATION** | **PRIMARY vs. ILLIT** | 0.702 | **0.049** | 0.494 | 0.999 | 0.798 | 0.157 | 0.585 | 1.090 |
|  | **SECONDARY vs. ILLIT** | 0.329 | **<0.001** | 0.215 | 0.502 | 0.702 | **0.044** | 0.498 | 0.991 |
| **HOUSEHOLD FACTORS** |  |  |  |  |  |  |  |  |  |
| **SOCIO-ECONOMIC** | **MEDIUM vs. LOW** | 1.082 | 0.590 | 0.811 | 1.444 | 0.733 | **0.013** | 0.575 | 0.936 |
|  | **HIGH vs. LOW** | 0.747 | 0.065 | 0.548 | 1.019 | 0.989 | 0.932 | 0.774 | 1.265 |
| **RESIDENTIAL AREA** | **RURAL vs. URBAN** | 0.924 | 0.551 | 0.712 | 1.199 | 0.672 | **<0.001** | 0.542 | 0.835 |
| **CROWDING** | **≥3 vs. <3 PER BEDR** | 1.983 | **<0.001** | 1.546 | 2.544 | 1.250 | **0.032** | 1.019 | 1.533 |
| **MONTHLY INCOME** | **≥1 vs. <1 BASKET** | 0.230 | **0.002** | 0.089 | 0.590 | 0.740 | 0.249 | 0.443 | 1.235 |
| **HOUSE CONSTRUCTION** | **CEMENT/BRICK vs. WOOD/BAMBOO** | 1.208 | 0.156 | 0.930 | 1.570 | 1.035 | 0.757 | 0.832 | 1.289 |
| **DOG IN HOUSE (BIRTH)** | **YES vs. NO** | 1.212 | 0.300 | 0.843 | 1.745 | 0.875 | 0.393 | 0.644 | 1.189 |
| **CAT IN HOUSE (BIRTH)** | **YES vs. NO** | 1.402 | 0.055 | 0.993 | 1.980 | 1.062 | 0.695 | 0.787 | 1.433 |
| **AGRICULTURE (TV)** | **YES vs. NO** | 1.495 | **0.002** | 1.163 | 1.920 | 0.881 | 0.216 | 0.720 | 1.077 |
| **BATHROOM (TV)** | **WC vs. latrine** | 0.609 | **0.001** | 0.449 | 0.826 | 1.139 | 0.303 | 0.889 | 1.460 |
| **HOUSEHOLD STH INFECTIONS** |  |  |  |  |  |  |  |  |  |
| **Mother** | **YES vs. NO** | 2.365 | **<0.001** | 1.839 | 3.042 | 1.634 | **<0.001** | 1.331 | 2.005 |
| **Father** | **YES vs. NO** | 3.200 | **<0.001** | 2.145 | 4.772 | 1.263 | 0.175 | 0.901 | 1.770 |
| **Siblings** | **YES vs. NO** | 2.473 | **<0.001** | 1.708 | 3.579 | 1.290 | 0.088 | 0.962 | 1.728 |
| **Any in household** | **YES vs. NO** | 1.925 | **<0.001** | 1.435 | 2.582 | 1.332 | **0.013** | 1.062 | 1.670 |

**S2 Table. Age-adjusted associations between child, maternal, and household factors with infections with any soil-transmitted helminth (STH) or protozoal parasite from 7 months to 8 years of age.**

Odds ratios (ORs) and 95% confidence intervals (CI) were estimated by fitting age, age2, and age3-adjusted longitudinal models using generalized estimating equations. Longitudinal binary outcomes were defined by presence/absence of any STH or any protozoa detected in stool samples from children during follow-up. Models were fit under missing completely at random assumption for unobserved data points. Characteristics are at time of birth of child (birth) or time-varying (TV) over the course of follow-up. STH—soil-transmitted helminth infections. Afro- Afro-Ecuadorian. Anthelmintic treatments—maternal reports of at least one anthelmintic treatment during the previous year. Monthly household income was classified according to receipt of an income sufficient to meet the basic needs of 4 persons (or 1 family basket) or US$480 in 2008. Household overcrowding was defined as 3 or more people per sleeping room. Agricultural exposures were defined by living on a farm or having at least weekly visits to a farm. Any infected with STH in the household represented any member of the child’s household with a positive stool sample collected around the time of birth of the cohort child.
