# Supplementary material for: Epidemiology of intestinal parasite infections and multiparasitism and their impact on growth and hemoglobin levels during childhood in tropical Ecuador: A longitudinal study using molecular detection methods
Source: PLoS Negl Trop Dis. 2025 Jun 16;19(6):e0013004. doi: 10.1371/journal.pntd.0013004 (PMC12169531; doi:10.1371/journal.pntd.0013004)
Supplement: S3 Table — Odds ratios (ORs) and 95% confidence intervals (CI) were estimated by fitting age, age2, and age3-adjusted longitudinal models using generalized estimating equations. Longitudinal binary outcomes were defined by presence/absence of any STH or any protozoa detected in stool samples from children during follow-up. Models were fit under missing completely at random assumption for unobserved data points. Characteristics are at time of birth of child (birth) or time-varying (TV) over the course of follow-up. STH—soil-transmitted helminth infections. Afro- Afro-Ecuadorian. Household overcrowding was defined as 3 or more people per sleeping room. Agricultural exposures were defined by living on a farm or having at least weekly visits to a farm. (DOCX) [file pntd.0013004.s003.docx]

| **Variables** | **Categories** | **Any STH** | | | | **Any protozoa** | | | |
| --- | --- | --- | --- | --- | --- | --- | --- | --- | --- |
|  |  | **OR** | **P-value** | **95%CI**  **low** | **95%CI**  **high** | **OR** | **P-value** | **95%CI low** | **95%CI high** |
| **CHILDHOOD FACTORS** |  |  |  |  |  |  |  |  |  |
| **Age** |  | **1.0064** | **<0.001** | **1.0052** | **1.0076** | **1.0055** | **<0.001** | **1.0046** | **1.0065** |
| **Age^2^** |  | **0.9999** | **0.001** | **0.9998** | **0.9999** | **0.9999** | **<0.001** | **0.9998** | **0.9999** |
| **Age^3^** |  | **1.0000** | **0.011** | **1.0000** | **1.0000** | **1.0000** | **<0.001** | **1.0000** | **1.0000** |
| **Sex** | **Female vs. male** |  |  |  |  | **0.710** | **0.002** | **0.571** | **0.883** |
| **Birth order** | **3^rd^-4^th^ vs. 1^st^-2^nd^** |  |  |  |  | 1.116 | 0.384 | 0.871 | 1.431 |
|  | **≥5^th^ vs. 1^st^-2^nd^** |  |  |  |  | **1.485** | **0.009** | **1.102** | **2.002** |
| **Delivery mode** | **Vaginal vs. Caesarean** | **0.652** | **0.035** | **0.438** | **0.970** | **0.743** | **0.022** | **0.576** | **0.958** |
| **Day care to 3 years** | **Yes vs. No** |  |  |  |  | **1.606** | **0.002** | **1.188** | **2.170** |
| **MATERNAL FACTORS** |  |  |  |  |  |  |  |  |  |
| **Ethnicity** | **Non-Afro vs. Afro** | **0.451** | **<0.001** | **0.321** |  |  |  |  |  |
| **HOUSEHOLD FACTORS** |  |  |  |  |  |  |  |  |  |
| **Socio-economic status** | **Medium vs. low** |  |  |  |  | **0.699** | **0.008** | **0.536** | **0.910** |
|  | **High vs. low** |  |  |  |  | 0.909 | 0.498 | 0.691 | 1.197 |
| **Area of residence** | **Rural vs. urban** |  |  |  |  | **0.717** | **0.008** | **0.560** | **0.917** |
| **Overcrowding** | **≥3 vs. <3** | **1.621** | **0.003** | **1.172** | **2.240** |  |  |  |  |
| **Agriculture (TV)** | **Yes vs. No** | **1.454** | **0.024** | **1.051** | **2.013** |  |  |  |  |
| **HOUSEHOLD STH INFECTIONS** |  |  |  |  |  |  |  |  |  |
| **Mother** | **Yes vs. No** | **2.008** | **<0.001** | **1.449** | **2.781** | **1.445** | **0.001** | **1.160** | **1.800** |

**S3 Table. Multivariable associations between child, maternal, and household factors with infections with any soil-transmitted helminth (STH) or protozoal parasite from 7 months to 8 years of age.**

Odds ratios (ORs) and 95% confidence intervals (CI) were estimated by fitting age, age2, and age3-adjusted longitudinal models using generalized estimating equations. Longitudinal binary outcomes were defined by presence/absence of any STH or any protozoa detected in stool samples from children during follow-up. Models were fit under missing completely at random assumption for unobserved data points. Characteristics are at time of birth of child (birth) or time-varying (TV) over the course of follow-up. STH—soil-transmitted helminth infections. Afro- Afro-Ecuadorian. Household overcrowding was defined as 3 or more people per sleeping room. Agricultural exposures were defined by living on a farm or having at least weekly visits to a farm.
