# Supplementary material for: Epidemiology of intestinal parasite infections and multiparasitism and their impact on growth and hemoglobin levels during childhood in tropical Ecuador: A longitudinal study using molecular detection methods
Source: PLoS Negl Trop Dis. 2025 Jun 16;19(6):e0013004. doi: 10.1371/journal.pntd.0013004 (PMC12169531; doi:10.1371/journal.pntd.0013004)
Supplement: S4 Table — Estimates are adjusted for weight or length at birth, respectively. Estimates (ORs) and 95% confidence intervals (CI) were estimated by fitting age, age2, age3, and age4-adjusted longitudinal models using generalized estimating equations. Models were fit under missing completely at random assumption for unobserved data points. (DOCX) [file pntd.0013004.s004.docx]

| **Variable** | **Categories** | **Weight-for-age (z-score)** | | | | **Height-for-age (z-score)** | | | |
| --- | --- | --- | --- | --- | --- | --- | --- | --- | --- |
|  |  | **Estimate** | **P-value** | **95% CI**  **low** | **95% CI high** | **Estimate** | **P-value** | **95% CI**  **low** | **95% CI**  **high** |
| **Any IPI** | **Yes vs. No** | **-0.197** | **<0.001** | **-0.305** | **-0.088** | **-0.180** | **0.001** | **-0.289** | **-0.070** |
| **Any STH** | **Yes vs. No** | **-0.279** | **<0.001** | **-0.406** | **-0.152** | **-0.224** | **0.001** | **-0.352** | **-0.095** |
| **Any protozoa** | **Yes vs. No** | **-0.134** | **0.013** | **-0.240** | **-0.029** | **-0.150** | **0.005** | **-0.257** | **-0.044** |
| **Multiparasitism** | **1 vs. 0** | **-0.147** | **0.014** | **-0.265** | **-0.029** | -0.109 | 0.074 | -0.229 | 0.011 |
|  | **≥2 vs. 0** | **-0.338** | **<0.001** | **-0.489** | **-0.188** | **-0.382** | **<0.001** | **-0.536** | **-0.228** |

**S4 Table. Age-adjusted associations for any parasite infection (IPI), any soil-transmitted helminth infection (STH), or any protozoa infection with longitudinal weight-for-age and height-for age z scores (g/dL). Estimates are adjusted for weight or length at birth, respectively.**

Estimates (ORs) and 95% confidence intervals (CI) were estimated by fitting age, age2, age3, and age4-adjusted longitudinal models using generalized estimating equations. Models were fit under missing completely at random assumption for unobserved data points.
