# Supplementary material for: Epidemiology of intestinal parasite infections and multiparasitism and their impact on growth and hemoglobin levels during childhood in tropical Ecuador: A longitudinal study using molecular detection methods
Source: PLoS Negl Trop Dis. 2025 Jun 16;19(6):e0013004. doi: 10.1371/journal.pntd.0013004 (PMC12169531; doi:10.1371/journal.pntd.0013004)
Supplement: S5 Table — Estimates show effects of current (measured at same time as hemoglobin) and previous (measured at previous time point) infections on longitudinal risk of anemia and average levels of hemoglobin. Estimates (ORs) and 95% confidence intervals (CI) were estimated by fitting age and age2-adjusted longitudinal models using generalized estimating equations. Models were fit under missing completely at random assumption for unobserved data points. (DOCX) [file pntd.0013004.s005.docx]

| **Parasite infection** | **Categories** | **Age-adjusted anaemia** | | | | **Age-adjusted hemoglobin** | | | |
| --- | --- | --- | --- | --- | --- | --- | --- | --- | --- |
|  |  | **OR** | **P-value** | **95% CI**  **low** | **95% CI high** | **Estimate** | **P-value** | **95% CI**  **low** | **95% CI high** |
| **Current infections** | | | | | | | | | |
| **Any IPI** | **Yes vs. No** | 1.219 | 0.232 | 0.881 | 1.686 | **-0.126** | **0.003** | **-0.209** | **-0.043** |
| **Any STH** | **Yes vs. No** | 1.009 | 0.968 | 0.642 | 1.585 | **-0.138** | **0.007** | **-0.238** | **-0.038** |
| **Any protozoa** | **Yes vs. No** | 1.227 | 0.220 | 0.885 | 1.700 | -0.078 | 0.058 | -0.158 | 0.003 |
| **Multiparasitism** | **1 vs. 0** | 1.290 | 0.152 | 0.911 | 1.828 | -0.125 | 0.015 | -0.226 | -0.025 |
|  | **≥2 vs. 0** | 1.061 | 0.828 | 0.621 | 1.812 | -0.053 | 0.417 | -0.182 | 0.075 |
|  | **≥2 vs. 1** | 0.822 | 0.483 | 0.476 | 1.421 | 0.072 | 0.281 | -0.059 | 0.203 |
| **Previous infections** | | | | | | | | | |
| **Any IPI** | **Yes vs. No** | 1.135 | 0.568 | 0.735 | 1.753 | **-0.124** | **0.009** | **-0.218** | **-0.031** |
| **Any STH** | **Yes vs. No** | 1.418 | 0.243 | 0.789 | 2.547 | **-0.195** | **0.001** | **-0.309** | **-0.081** |
| **Any protozoa** | **Yes vs. No** | 1.107 | 0.653 | 0.711 | 1.723 | **-0.118** | **0.013** | **-0.212** | **-0.025** |

**S5 Table. Age-adjusted associations for any parasite infection (IPI), any soil-transmitted helminth infection, or any protozoa infection with longitudinal risk of anemia and hemoglobin levels (g/dL) between 7 months and 8 years of age. Estimates show effects of current (measured at same time as hemoglobin) and previous (measured at previous time point) infections on longitudinal risk of anemia and average levels of hemoglobin.**

Estimates (ORs) and 95% confidence intervals (CI) were estimated by fitting age and age2-adjusted longitudinal models using generalized estimating equations. Models were fit under missing completely at random assumption for unobserved data points.
