# Supplementary material for: Epidemiology of intestinal parasite infections and multiparasitism and their impact on growth and hemoglobin levels during childhood in tropical Ecuador: A longitudinal study using molecular detection methods
Source: PLoS Negl Trop Dis. 2025 Jun 16;19(6):e0013004. doi: 10.1371/journal.pntd.0013004 (PMC12169531; doi:10.1371/journal.pntd.0013004)
Supplement: S6 Table — Estimates show effects of current (measured at same time as eosinophilia) and previous (measured at previous time point) infections on longitudinal risk of eosinophilia. (DOCX) [file pntd.0013004.s006.docx]

| **Parasite infection** | **Categories** | **Age-adjusted eosinophilia** | | | |
| --- | --- | --- | --- | --- | --- |
|  |  | **OR** | **P-value** | **95%CI**  **low** | **95%CI**  **high** |
| **Current infections** | | | | | |
| **Any IPI** | **Yes vs. No** | **1.640** | **<0.001** | **1.273** | **2.113** |
| **Any STH** | **Yes vs. No** | **2.253** | **<0.001** | **1.722** | **2.947** |
| **Any protozoa** | **Yes vs. No** | **1.327** | **0.019** | **1.048** | **1.679** |
| **Previous infections** | | | | | |
| **Any IPI** | **Yes vs. No** | 1.258 | 0.081 | 0.972 | 1.629 |
| **Any STH** | **Yes vs. No** | **1.389** | **0.031** | **1.031** | **1.873** |
| **Any protozoa** | **Yes vs. No** | 1.052 | 0.701 | 0.814 | 1.358 |

**S6 Table. Age-adjusted associations for any parasite infection (IPI), any soil-transmitted helminth infection, or any protozoa infection with longitudinal risk of eosinophilia between 7 months and 8 years of age. Estimates show effects of current (measured at same time as eosinophilia) and previous (measured at previous time point) infections on longitudinal risk of eosinophilia.**

Estimates (ORs) and 95% confidence intervals (CI) were estimated by fitting age and age2-adjusted longitudinal models using generalized estimating equations. Models were fit under missing completely at random assumption for unobserved data points.
