# Supplementary figures and images for: Epidemiology of intestinal parasite infections and multiparasitism and their impact on growth and hemoglobin levels during childhood in tropical Ecuador: A longitudinal study using molecular detection methods
Source: PLoS Negl Trop Dis. 2025 Jun 16;19(6):e0013004. doi: 10.1371/journal.pntd.0013004 (PMC12169531; doi:10.1371/journal.pntd.0013004)

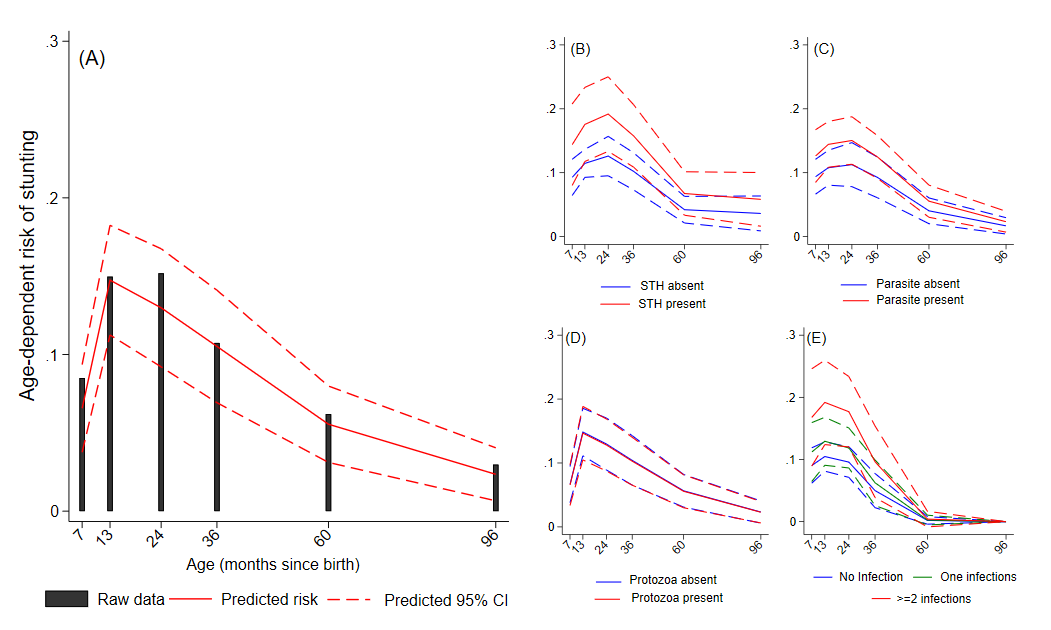

Supplement: S1 Fig — A – All children. B - Any soil-transmitted helminth (STH) parasite. C – Any intestinal parasite (Parasite) infection. D - Any intestinal protozoal (Protozoa) parasite. E – Multiparasitism. Y axes show proportions with stunting (defined as height-for-age z scores <=2). Interrupted curves represent 95% confidence intervals. (TIF) [file pntd.0013004.s008.tif]

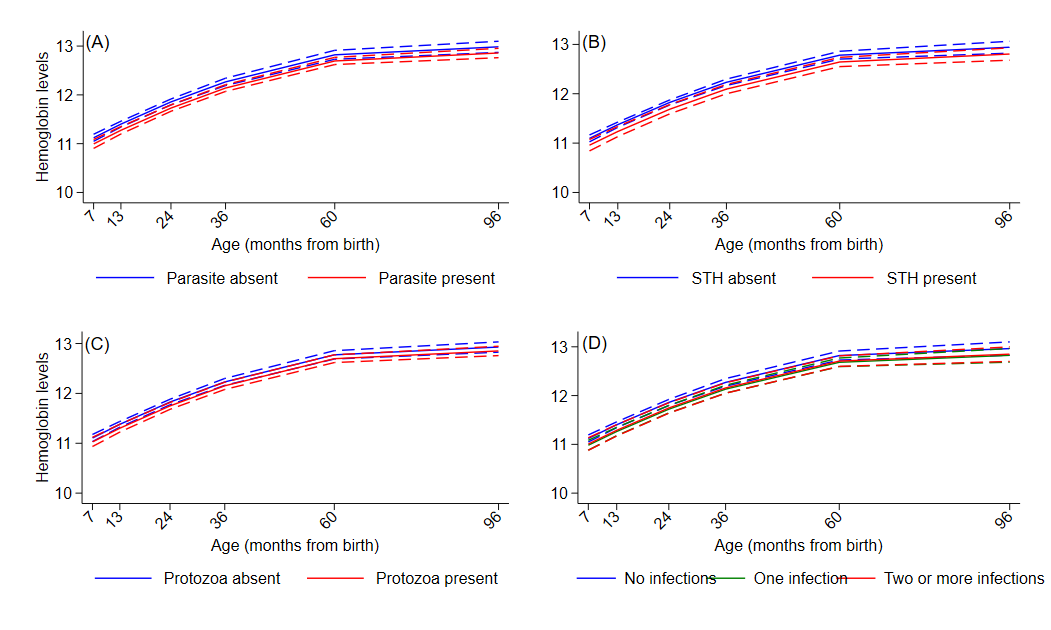

Supplement: S2 Fig — A – Any intestinal parasite (Parasite) infection. B- any soil-transmitted helminth (STH) parasite. C – any intestinal protozoal (Protozoa) parasite. D – Multiparasitism. Y axes show hemoglobin (g/dL). Interrupted curves represent 95% confidence intervals. (TIF) [file pntd.0013004.s009.tif]

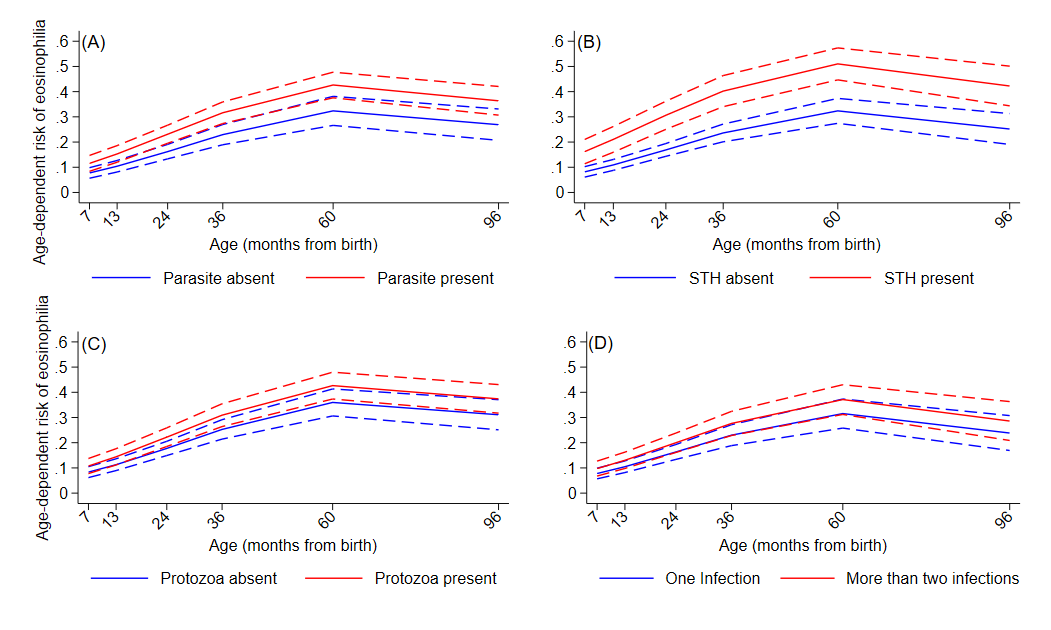

Supplement: S3 Fig — A – Any intestinal parasite (Parasite) infection. B- any soil-transmitted helminth (STH) parasite. C – any intestinal protozoal (Protozoa) parasite. D – Multiparasitism. Y axes show proportions with eosinophilia (defined as>= 500 cells/μL). Interrupted curves represent 95% confidence intervals. (TIF) [file pntd.0013004.s010.tif]
