# Supplementary material for: Epidemiology of intestinal parasite infections and multiparasitism and their impact on growth and hemoglobin levels during childhood in tropical Ecuador: A longitudinal study using molecular detection methods
Source: PLoS Negl Trop Dis. 2025 Jun 16;19(6):e0013004. doi: 10.1371/journal.pntd.0013004 (PMC12169531; doi:10.1371/journal.pntd.0013004)
Supplement: Attachment — Submitted filename: Point_by_point_response_PLoS_NTD.docx [file pntd.0013004.s012.docx]

'Response to Reviewers'

Abstract:
Methods - rephrase "studies for 8 years"

Response: We have rephrased for clarity as follows: “….Ecuadorian birth cohort followed-up to 8 years of age”

Results - Greater parasite numbers suggests intensity to me perhaps rephrase for clarity

Response: We have rephrased for clarity as follows: “….greater effects with greater number of parasite species”

Methods:
Maternal questionnaires were repeated when the child was... p. 8

Response: We have rephrased as suggested: “Maternal questionnaires were repeated when the child was 7 and 13 months, and 2, 3, 5, and 8 years of age

Rephrase : Fit back" p. 11

Response: We have rephrased for clarity as follows: “The final most parsimonious model using a complete data sample was then fitted back to the original data on as many observations as possible”.

Discussion:
Other systemic helminths were absent - please clarify p. 27

Response: We have rephrased for clarity as: “The study of a population in a setting where systemic helminth (e.g., trematode and filarial worms) and protozoal pathogens (e.g. malaria) that may themselves modify childhood growth and nutrition are largely absent, allowed us to more clearly evaluate the effects of enteric polyparasitism on study outcomes”.

Reviewer #1: The manuscript presents a comprehensive longitudinal study on the epidemiology of intestinal parasite infections (IPIs) and multiparasitism in a birth cohort from tropical Ecuador, utilizing molecular detection methods. The study addresses an important gap in understanding the impact of IPIs on childhood growth and hemoglobin levels. The findings are relevant to public health, particularly in endemic regions, and align with WHO priorities for reducing morbidity associated with parasitic infections. The study design is robust, and the use of molecular methods enhances the sensitivity of parasite detection. However, some areas require clarification or improvement to strengthen the manuscript.

1. Please use updated data and valid references for statistics on IPI-related prevalence and DALYs presented in the introduction.

Response: We have updated crude estimates for IPI-related prevalence and the associated references as follows on page 5: “Intestinal parasitic infections were estimated to infect almost 2 billion humans in 2019 [5]. Approximately 1 billion were estimated to be infected with STH parasites causing 1.9 million DALYs [6], and up to 300 millions suffer severe morbidity [7].”

2. Please clarify whether qPCR testing was performed on all samples at each time point or only on those that were microscopy-positive.

Response: qPCR was done on all samples. We have clarified this on page 11: “All stool samples were analyzed using qPCR irrespective of the results of microscopy, and the only results of qPCR analyses were used in this analysis.”

3. Please clarify how other unmeasured potential confounders (e.g., dietary intake, micronutrient supplementation) that could influence growth and hemoglobin outcomes might affect the results of this study.

Response: We have now included specific examples of unmeasured potential confounders and discussed their potential effects on the results as follows: “Potential unmeasured confounders include micronutrient supplementation and dietary quality both of which could affect nutritional outcomes, but which have less clear effects on longitudinal trajectories of IPI risk.”

4. The lack of a significant effect on anemia (despite lower hemoglobin levels) warrants further discussion. Could this be due to the low prevalence of hookworm (a major driver of anemia) in the cohort?

Response: We have included a sentence on this in the Discussion as requested as follows: “As for stunting, the prevalence of anemia was probably too low to detect significant effects of IPIs on this outcome - the low prevalence of hookworm, a major cause of anemia in STH-endemic populations, would have further limited anemia risk.”

Reviewer #2: The authors should be commended on this powerful study with meticulous detail throughout. In particular, the major strength of the study is its longitudinal design covering child age from early childhood to school age. Consequently the impact of parasitism is clearly shown in contrast to short-term follow-up studies that mainly informed recent meta-analyses. Furthermore, the use of sensitive molecular detection methods allowed the identification of low intensity infections. As pointed out by the authors, the impact of specific parasite species was not possible but this study highlights the methodology in which future studies can be designed to delineate such species-specific impacts.

Response: We thank the reviewer for these positive comments.
